# RS-STGCN: Regional-Synergy Spatio-Temporal Graph Convolutional Network for emotion recognition

**DOI:** 10.3389/fnins.2025.1704476

**Published:** 2025-12-05

**Authors:** Yunqi Han, Yifan Chen, Hang Ruan, Deqing Song, Haoxuan Xu, Haiqi Zhu

**Affiliations:** 1 Faculty of Computer Science and Information Technology, University Putra Malaysia, Serdang, Selangor, Malaysia; 2 School of Computer Science, University of Nottingham Malaysia, Semenyih, Malaysia; 3 Faculty of Computing, Harbin Institute of Technology, Harbin, China; 4 School of Medicine and Health, Harbin Institute of Technology, Harbin, China

**Keywords:** emotion recognition, electroencephalography, spatio-temporal graph convolutional network, dynamic graph construction, functional connectivity

## Abstract

Decoding emotional states from electroencephalography (EEG) signals is a fundamental goal in affective neuroscience. This endeavor requires accurately modeling the complex spatio-temporal dynamics of brain activity. However, prevailing approaches for defining brain connectivity often fail to reconcile predefined neurophysiological priors with task-specific functional dynamics. This paper presents the Regional-Synergy Spatio-Temporal Graph Convolutional Network (RS-STGCN), a novel framework designed to bridge this gap. The core innovation is the Regional Synergy Graph Learner (RSGL), which integrates known physiological brain-region priors with a task-driven optimization process. It constructs a sparse, adaptive graph by modeling connectivity at two distinct levels. At the intra-regional level, it establishes core information backbones within functional areas. This ensures efficient and stable local information processing. At the inter-regional level, it adaptively identifies critical, sparse long-range connections. These connections are essential for global emotional integration. This dual-level, dynamically learned graph then serves as the foundation for the spatio-temporal network. This network effectively captures evolving emotional features. The proposed framework demonstrates superior recognition accuracy, achieving state-of-the-art results of 88.00% and 85.43% on the public SEED and SEED-IV datasets, respectively, under a strict subject-independent protocol. It also produces a neuroscientifically interpretable map of functional brain connectivity, identifying key frontal-parietal pathways consistent with established attentional networks. This work offers a powerful computational approach to investigate the dynamic network mechanisms underlying human emotion, providing new data-driven insights into functional brain organization. The code and datasets are available at https://github.com/YUNQI1014/RS-STGCN.

## 1 Introduction

Decoding the neural bases of human emotion is a central goal in affective neuroscience (Wu et al., 2022). This research advances core theories in psychology and cognitive science. It also helps provide objective biomarkers for affective disorders (Davidson and McEwen, 2012). Electroencephalography (EEG) is an ideal tool for this purpose. Its high temporal resolution effectively captures the neural dynamics of emotional states (Coan and Allen, 2007). However, decoding emotions from EEG signals remains a significant challenge. These signals are high-dimensional, noisy, and vary greatly across subjects (Jenke et al., 2014; Erichsen et al., 2024).

Recent neuroscience research indicates that emotions arise from large-scale brain network interactions (Lindquist et al., 2012; Kober et al., 2008). Functional connectivity is a key measure used to quantify these neural interactions (Friston, 2011). Changes in functional connectivity patterns often correspond to different cognitive and emotional states (Vytal and Hamann, 2010). Therefore, graph-based models provide a powerful framework for this analysis. In these models, EEG electrodes are represented as nodes and their functional connections as edges (Bullmore and Sporns, 2009; Rubinov and Sporns, 2010). This approach effectively models the brain's functional architecture during emotion processing.

Graph Convolutional Networks (GCNs) show significant promise for analyzing these brain graphs (Kipf and Welling, 2017; Zhong et al., 2020). They have been successfully applied to EEG-based emotion recognition (Song et al., 2018; Xu et al., 2025). However, GCN performance critically depends on the quality of the input graph structure, a foundational challenge in graph-based modeling for complex systems (Defferrard et al., 2016; Chen et al., 2025). Recent research has advanced EEG signal analysis through both deep learning and graph-based frameworks. (Aboalsamh and Al-Haddad 2022) proposed an emotion detection approach based on zero-time windowing-based epoch estimation and relevant electrode identification, improving the reliability of EEG feature extraction. EEG-GNN applied a graph neural model that integrated spectral and spatial information between electrodes, improving the reliability of EEG classification (Demir et al., 2021). EEG-GAT extended this framework by incorporating attention mechanisms to capture cross-regional dependencies more effectively (Demir et al., 2022). EEG-GCNN introduced a domain-guided graph convolutional model that enhanced interpretability and diagnostic accuracy in brain network analysis (Wagh and Varatharajah, 2020). In parallel, Hjorth feature analysis improved EEG representation by introducing concise temporal descriptors for biomedical interpretation (Alawee et al., 2023). These studies demonstrate steady progress in EEG modeling, although most still depend on fixed or heuristic graph definitions that limit their capacity to represent dynamic emotional processes.

Current graph construction methods fall into two primary yet suboptimal directions. One approach uses static graphs based on physical distance or predefined connectivity measures (Li et al., 2018b; Vicente et al., 2011; Dev et al., 2024). While grounded in physiology, these static graphs fail to capture task-specific neural dynamics (Hutchison et al., 2013). This limitation often results in suboptimal classification performance (Li et al., 2018b). An alternative approach learns the graph structure directly from the data (Veličković et al., 2018; Li R. et al., 2018). This includes diverse methods. For instance, IIR-AGCN (Xu et al., 2024) uses an “intra-inter region” concept based on self-attention, but it is designed for skeleton-based action recognition. This domain involves articulated physical structures, which is different from functional brain networks. Other powerful, general-purpose learners like the Differentiable Graph Module(DGM) (Kazi et al., 2022), learn the graph end-to-end. However, these methods, whether general or domain-specific, often lack neurophysiological constraints. As they are optimized solely for the downstream task, they may learn spurious connections and exhibit poor generalisability (Kazi et al., 2022). This creates a fundamental conflict. The gap between flexible, task-driven learning such as DGM and rigid physiological priors from static graphs remains unresolved.

This situation presents a fundamental challenge. A principled method is needed to effectively integrate neurophysiological priors with task-driven graph optimization. This core limitation persists even in more advanced architectures. For instance, Spatio-Temporal Graph Convolutional Networks (STGCNs) are designed to jointly model spatial and temporal dynamics (Yu et al., 2018; Tsai and Chen, 2023). However, their performance is still constrained by the same flawed spatial graph definitions. This unresolved issue limits both the accuracy and the neuroscientific interpretability of current state-of-the-art models.

To bridge this gap, this paper introduces the RS-STGCN. The model's core innovation is the Regional Synergy Graph Learner (RSGL) module. The RSGL integrates known brain functional region priors with a task-driven optimization process. It constructs a sparse and adaptive graph using a two-level strategy. At the intra-regional level, a Minimum Spanning Tree (MST) establishes an efficient information backbone within each brain region. At the inter-regional level, a budgeted selection mechanism identifies the most critical long-range connections, which are crucial for emotion processing. This dynamically learned graph provides a robust foundation for the spatio-temporal network, effectively capturing the evolving features characteristic of emotional states.

This work presents a novel framework that uniquely addresses the limitations of both static graphs and unconstrained graph learning methods. The central contribution is the RSGL, a neurophysiologically-inspired graph learner that models brain connectivity at two synergistic levels: stable intra-regional backbones and adaptive inter-regional pathways. This dual-level representation is integrated into an end-to-end spatio-temporal network. The framework not only achieves state-of-the-art accuracy but also produces interpretable connectivity maps, offering data-driven insights into functional brain organization during emotion processing.

The primary contributions of this work are as follows:

1. **Regional Synergy Graph Learner:** A novel graph construction module that models brain connectivity at two levels. It reconciles neurophysiological priors via intra-regional backbones with task-driven optimization via inter-regional connection selection.
2. **End-to-End Spatio-Temporal Framework:** A unified RS-STGCN framework that integrates the adaptive graph learner with a spatio-temporal network. It achieves superior performance on benchmark EEG emotion recognition tasks.
3. **Interpretable Brain Network Dynamics:** A data-driven approach that produces neuroscientifically plausible functional connectivity maps. These maps provide interpretable evidence of the dynamic network mechanisms underlying human emotion.

## 2 Method

### 2.1 Concepts and definitions

The EEG-based emotion recognition is modeled as a node classification problem on a dynamic graph. In the graph, each node represents an EEG electrode channel, and edges indicate potential functional connections between different brain functional regions. The graph structure evolves dynamically during training to reflect task-driven spatial dependencies and physiological priors related to emotional states.

**Node features:** Let $X_{i}\in {\mathbb{R}}^{N\times F}$ be the node feature matrix at time step t, where *N* represents the number of EEG electrodes, and *F* represents the differential entropy (DE) feature calculated based on five typical frequency bands (delta band: 0–4 Hz, theta band: 4–8 Hz, alpha band: 8–13 Hz, beta band: 13–30 Hz, gamma band: 30–45 Hz). This feature can characterize the spectral characteristics of EEG signals and is highly correlated with emotional state.

**Dynamic adjacent matrix:** Let $A_{t}\in {\mathbb{R}}^{N\times N}$ be the adjacency matrix at time step *t*, which characterizes the strength or existence of functional connections between electrode nodes. The adjacency matrix *A*_*t*_ is dynamically generated by the RSGL. It combines physiological priors with adaptive cross-functional region connectivity to achieve a sparse and biologically meaningful brain functional connectivity map.

By jointly utilizing node features *X*_*i*_ and adjacency matrix *A*_*t*_, the constructed dynamic graph representation can simultaneously capture local neural activity patterns and long-range functional interactions, providing support for the recognition of complex emotional states.

### 2.2 Regional-Synergy Spatio-Temporal Graph Convolutional Network

**Figure 1:**
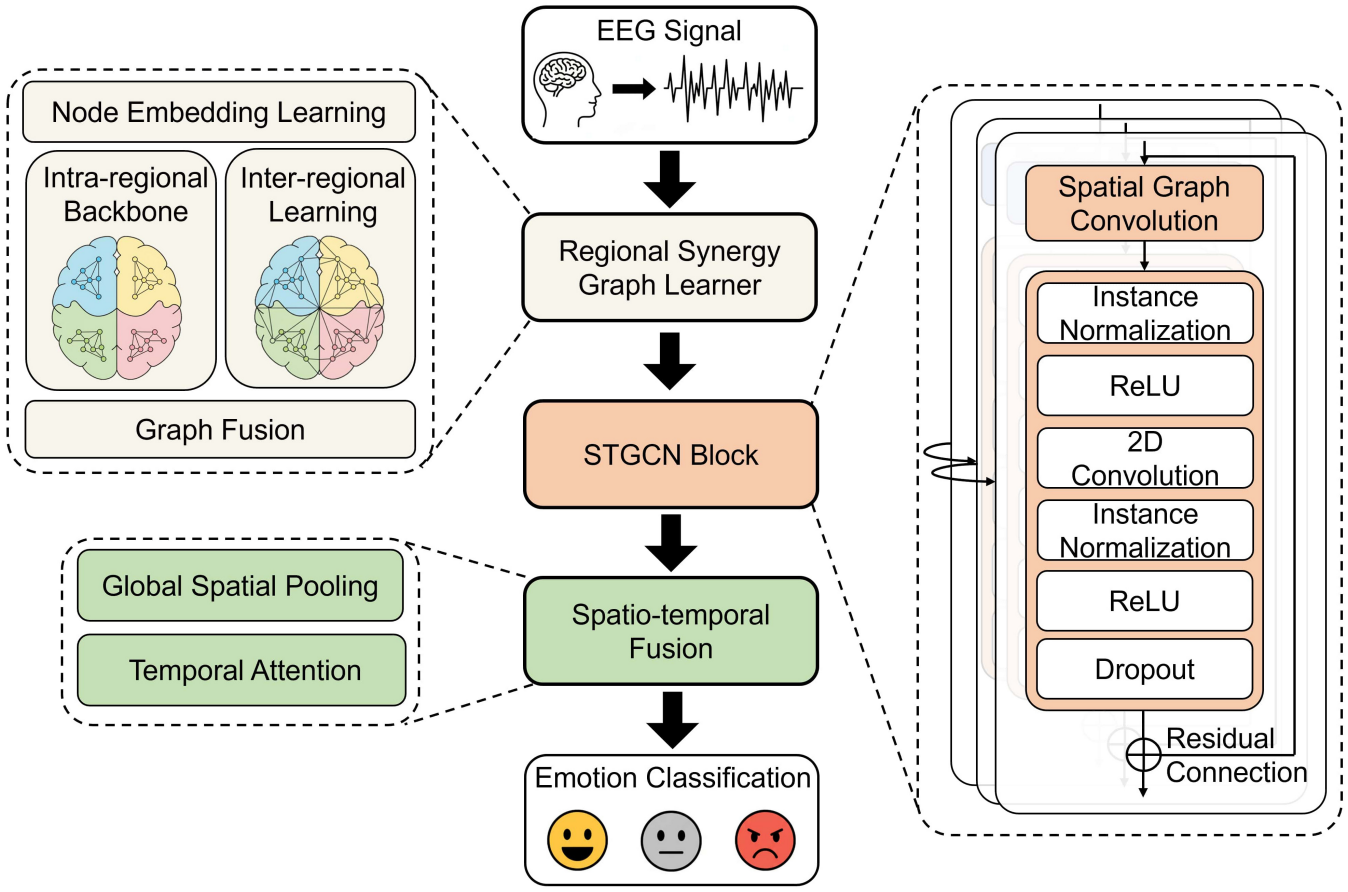
The overall framework of the proposed RS-STGCN model.

The Proposed RS-STGCN is a framework designed for EEG-based emotion recognition. Its primary objective is to learn a task-specific functional connectivity graph that is constrained by neurophysiological priors. The framework comprises three core components that are jointly optimized: an adaptive graph learner generating the adjacency matrix *A*, a spatio-temporal encoder extracting the feature *H*, and a final emotion classifier. The overall architecture is illustrated in Figure 1, which shows how these modules are integrated into a unified pipeline. This integrated architecture is specifically designed to produce a sparse and interpretable graph, thereby enhancing biological plausibility and classification performance. The complete training procedure is detailed in Algorithm 1.

Algorithm 1

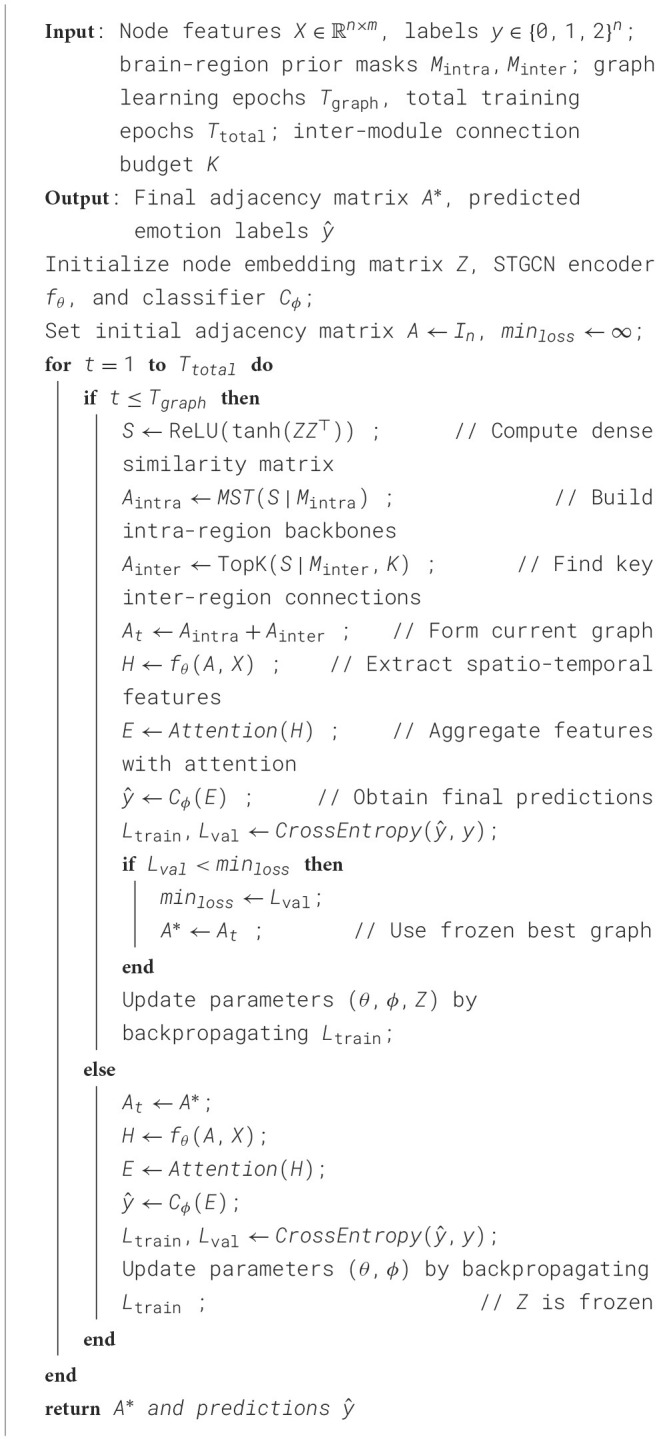

#### 2.2.1 Regional Synergy Graph Learner

The RSGL is a core component designed to construct a sparse, adaptive, and neuroscientifically plausible brain graph. The process is guided by neurophysiological priors and optimized in a task-driven manner. The graph construction is decomposed into three stages: defining the graph's foundational structure based on brain functional regions, modeling intra-regional connectivity, and identifying salient inter-regional connections.

##### 2.2.1.1 Neurophysiological priors and graph parameterization

The foundation of the RSGL is built upon the Neurophysiological organization of the brain, where EEG electrodes are grouped according to their corresponding cortical regions, such as the frontal, temporal, and occipital lobes. The foundation of the RSGL is built upon the neurophysiological organization of the brain. This partitioning of EEG electrodes is guided by the principle of functional specialization. This principle posits that the brain is organized into distinct, functionally coherent regions (Zhu et al., 2025; Kanwisher, 2010; Rubinov and Sporns, 2010). Grouping EEG channels based on these cortical areas is a common and effective practice in graph-based brain network analysis (Zhong et al., 2020).

##### 2.2.1.2 Neurophysiological priors and graph parameterization

The foundation of the RSGL is built upon the neurophysiological organization of the brain, specifically the grouping of EEG electrodes by cortical region. This partitioning is a critical prior for the model. Following the international 10-20 system for electrode placement (Klem, 1999), the 62 EEG channels in this study are grouped into five distinct functional regions: frontal, temporal, central, parietal, and occipital. This partitioning is visualized in the RS-STGCN brain graph. This approach is guided by the principle of functional specialization (Zhu et al., 2025; Kanwisher, 2010; Rubinov and Sporns, 2010). Grouping channels by cortical area is a common and effective practice in graph-based brain network analysis (Zhong et al., 2020).

The regional decomposition of EEG electrodes follows the formal definition in Equation 1, ensuring that each electrode belongs exclusively to one functional brain region.This partitioning reflects the principle of functional specialization in the brain (Kanwisher, 2010; Rubinov and Sporns, 2010). The set of all *N* electrode nodes *V* is formally partitioned into *R* disjoint regional subsets:


$$\begin{array}{l} V=\overset{R}{\underset{r=1}{⋃}}V_{r},\;V_{r}\cap V_{s}=\emptyset \;\left(r\neq s\right) \end{array}$$ (1)

Where *V*_*r*_, *V*_*s*_ represents the set of electrodes belonging to the *r*−*th* brain region, *v* represents the set of all nodes in the graph. *R* is the number of brain functional regions, and ${⋃}_{r=1}^{R}V_{r}$ represents the union of all electrode sets for all brain functional regions. *V*_*r*_∩*V*_*s*_ = ∅ (*r*≠*s*) indicates that each electrode node belongs to only one brain region.

To enable task-driven graph learning, the learnable node embedding matrix *Z*∈ℝ^*n*×*d*^ is introduced, where n is the number of EEG channels and d is the preset embedding dimension. The connections within the brain functional region are established using the similarity matrix *S*, which is calculated from the node embedding *Z*∈ℝ^*n*×*d*^:


$$\begin{array}{l} S=\mathrm{ReLU}\left(\tanh\left(ZZ^{⊤}\right)\right) \end{array}$$ (2)

In Equation 2, the inner product *ZZ*^⊤^ calculates the similarity between the node embeddings. The tanh function stabilizes the values, while the ReLU function ensures the resulting affinity scores are non-negative. The matrix *S* represents a fully connected graph of potential functional connections, which requires further refinement to achieve sparsity and structural coherence.

##### 2.2.1.3 Intra-regional backbone construction

To model core information pathways, a sparse and connected backbone is built for each brain region. This approach is motivated by the neuroscientific principle of efficient local processing. For each regional subgraph defined by the nodes *V*_*r*_, a MST is extracted. This is based on the corresponding local similarity scores in *S*. The MST connects all nodes within the region without forming cycles. It also maximizes the total edge weight. From a theoretical standpoint, this intra-regional backbone construction also provides structural stability across folds and random seeds. Because the MST algorithm deterministically yields a unique solution for each regional similarity matrix, the resulting subgraphs preserve identical topological patterns when the same data distribution is used. This property ensures that the learned intra-regional connectivity remains consistent and reproducible throughout training, thereby forming a stable foundation for subsequent inter-regional graph learning. The intra-regional backbone construction strictly follows Equation 3, where the MST operation guarantees sparse yet fully connected subgraphs within each functional region.This operation is formulated as:


$$\begin{array}{l} A_{intra}^{\left(r\right)}=\text{MST}\left(S\left[V_{r},V_{r}\right]\right) \end{array}$$ (3)

where *S*[*V*_*r*_, *V*_*r*_] is the submatrix of *S* containing similarities among nodes in region *r*. Since standard algorithms compute a MST, the operation is performed on negated similarity scores to find the maximum. The final intra-regional adjacency matrix, *A*_*intra*_, is the aggregation of all such backbones from every region. This process ensures that a highly connected network exists within each functional module.

##### 2.2.1.4 Inter-regional connection learning

Studies have shown that emotion-related brain activity often involves the synergy of multiple brain functional regions. These inter-regional connections are often few and sparse, [citation needed]. To capture these critical long-range dependencies, the RSGL employs a budgeted-TopK selection strategy. This approach identifies the most salient connections between distinct brain functional areas:


$$\begin{array}{l} A_{\text{inter}}=\text{TopK}\left(S⊙M_{\text{inter}},K\right) \end{array}$$ (4)

In Equation 4, ⊙ represents the element-wise Hadamard product, *M*_*inter*_ is the cross-functional region mask matrix, ensuring that only cross-region candidate edges are considered, and *K* is the global hyperparameter of the cross-module connection budget to ensure that only the representative long-range dependency edges are retained in the end.

##### 2.2.1.5 Final graph fusion

Through the backbone structure within the above integrated module and the bridging edges across functional areas, a complete graph is obtained:


$$\begin{array}{l} A=A^{intra}+A^{inter} \end{array}$$ (5)

The resulting graph *A* is both sparse and structurally meaningful. As shown in Equation 5, the final synergy graph is formed by fusing intra-regional and inter-regional connections into a unified adjacency matrix, providing the structural foundation for the subsequent STGCN module. From a theoretical perspective, the intra-regional subgraph construction based on the MST algorithm guarantees deterministic and reproducible topology. Given that MST always yields a unique connected structure for a given similarity matrix, the local connectivity patterns within each functional region remain stable across different folds and random seeds. This design ensures that the learned graph preserves essential intra-regional relationships without introducing random structural variations. It incorporates neurophysiological priors and simultaneously optimized for the specific task of emotion recognition. This learned graph then serves as the foundational structure for the STGCN module.

#### 2.2.2 STGCN: Spatial-Temporal Graph Convolutional Network

The STGCN block is the fundamental unit for learning spatio-temporal representations. It consists of a spatial graph convolution module followed by a temporal convolution module, integrated within a residual connection.

##### 2.2.2.1 Spatial graph convolution module

The spatial graph convolution module is designed to capture patterns of functional connectivity between EEG channels. It employs a standard GCN that operates on the graph structure *A* learned by the RSGL. Crucially, this operation is applied independently to the feature representation of each time step, allowing the model to learn time-varying spatial patterns. The propagation rule for a single GCN layer is defined as:


$$\begin{array}{l} H^{\left(l+1\right)}\;=\;\sigma \left(\tilde{A}H^{\left(l\right)}W^{\left(l\right)}\right) \end{array}$$ (6)

Here, *H*^(*l*)^ is the feature matrix at layer *l*, *W*^(*l*)^ is a learnable weight matrix, and σ is a non-linear activation function. As expressed in Equation 6, spatial dependencies among EEG channels are aggregated through the learned adjacency matrix Ã, enabling the extraction of task-relevant spatial patterns.The matrix Ã is the symmetrically normalized adjacency, which is crucial for stabilizing the learning process. It is computed as:


$$\begin{array}{l} \tilde{A}\;=\;D^{-1/2}AD^{-1/2} \end{array}$$ (7)

The normalization strategy in Equation 7, where *D* is the diagonal degree matrix of *A*, with ${\tilde{D}}_{ii}=\underset{j}{\sum }{Ã}_{ij}$. This normalization prevents the scale of feature representations from changing during graph convolutions.

##### 2.2.2.2 Temporal convolution module

Following the spatial feature aggregation, the resulting representation *H*_*s*_ serves as the input to the temporal convolution module. The tensor $H_{s}\in {\mathbb{R}}^{B\times F_{in}\times C\times T}$ encapsulates the spatially-aware node features at every time step, where *B* is the batch size, *F*_*in*_ is the input feature dimension, *C* is the number of nodes, and *T* is the number of time steps.

The core of this module is a TCN-based block designed to extract dynamic patterns. The main processing path is a carefully defined sequence of operations. As shown in Figure 1, the path of the temporal convolution module begins with an Instance Normalization layer and a ReLU activation. This is followed by the central 2D convolution, which performs the temporal feature extraction. The sequence concludes with another set of Instance Normalization, ReLU activation, and a Dropout layer for regularization. This multi-step design allows for the robust extraction of temporal features while stabilizing the training process.

A crucial component of the architecture is a residual connection, which adds the module's input *H*_*s*_ to the output of the main temporal processing path to ensure stable gradient flow. A residual mapping *r*(·), 0 is used to align the tensor shapes if the number of features or the temporal dimension changes. This mapping is precisely defined as:


$$r(H_{s})=\{\begin{array}{ll} H_{s}, & \text{if }F_{\text{in}}=F_{\text{out}}\text{ and stride}=1,\\ {\text{Conv}}_{1\times 1}(H_{s}), & \text{otherwise.} \end{array}$$ (8)

In Equation 8, the *Conv*_1 × 1_ operation is a standard 1 × 1 convolution, which functions as a linear projection to match the channel dimension *F*_*in*_ *to F*_*out*_ without altering the temporal length. Let TCN (*H*_*s*_) denote the output of the main temporal processing path described above. The final output of the entire STGCN block, *H*_*block*_ is obtained by fusing the two paths and applying a non-linear activation:


$$\begin{array}{l} H_{block}=\text{ReLU}\left(\text{TCN}\left(H_{s}\right)+r\left(H_{s}\right)\right) \end{array}$$ (9)

This residual fusion improves the stability of the time-series modeling and allows the network to effectively learn deep spatio-temporal features.

##### 2.2.2.3 Spatio-temporal fusion

After the input data is processed by the stack of STGCN blocks, the resulting high-level feature tensor, $H_{L}\in {\mathbb{R}}^{B\times F_{in}\times C\times T}$, need to be aggregated into a single feature vector for classification. This is accomplished through a two-stage process. This is achieved through a two-step process: global pooling followed by temporal attention. First, a global average pooling operation is performed across the spatial dimension. This operation averages the feature representations of all nodes at each time step, producing a unified sequence representation *H*_*p*_ that preserves only the global temporal dynamics. The operation is defined as:


$$\begin{array}{l} H_{p}=\frac{1}{C}\overset{C}{\underset{i=1}{\sum }}H_{L,i} \end{array}$$ (10)

Here, *H*_*L, i*_ denotes the feature tensor of the c-th node from the final block's output *H*_*L*_. The resulting $H_{p}\in {\mathbb{R}}^{B\times F_{L}\times T}$ summarizes the overall brain state at each moment. Next, a temporal attention mechanism is applied to *H*_*p*_ to adaptively weigh the importance of features at different moments in time. Given the sequence of feature vectors $h_{t}\in {\mathbb{R}}^{F_{L}}$ at each time step t from *H*_*p*_, the mechanism operates as follows. First, an unnormalized attention score *S*_*t*_ is computed:


$$\begin{array}{l} s_{t}=v^{T}\tanh\left(Wh_{t}+b\right) \end{array}$$ (11)

where *W*, *b*, and *v* are are learnable weight and bias parameters of the attention network. These scores are then normalized using the Softmax function to produce the final attention weights *a*_*t*_:


$$\begin{array}{l} a_{t}=\;\frac{exp\left(s_{t}\right)}{\sum_{\tau =1}^{T}exp\left(s_{\tau }\right)} \end{array}$$ (12)

The final global feature representation E is computed as the weighted sum of the temporal features:


$$\begin{array}{l} E=\overset{T}{\underset{t=1}{\sum }}a_{t}h_{t}. \end{array}$$ (13)

Finally, the resulting feature vector $E\in {\mathbb{R}}^{B\times F_{L}}$ is passed through a fully connected linear layer with a Softmax activation to produce the final class probabilities.

In summary, the STGCN architecture enables a deep and hierarchical fusion of information from both the spatial and temporal domains. Within each block, the graph convolution captures spatial dependencies across the learned brain graph, while the subsequent temporal convolution models the local evolution of these features over time. By stacking these blocks, the network learns progressively abstract spatio-temporal representations, moving from local patterns to more global dynamics. The fusion module then distills these learned features into a representation that encapsulates the essential dynamic brain network patterns for emotion classification.

### 2.3 Neuroscientific relevance of the proposed framework

The RS-STGCN framework connects computational modeling with neuroscientific interpretation. Its graph learning process is designed to optimize classification accuracy while maintaining biologically meaningful connectivity. The RSGL captures stable intra-regional coherence, whereas the spatio-temporal encoder models dynamic inter-regional communication. Together, these components align with recognized cortical interactions, including frontal–parietal and frontal–occipital pathways involved in emotion regulation. This alignment strengthens the framework's interpretability and ensures consistency with neurophysiological evidence.

### 2.4 Model training and optimization

The model's parameters are optimized end-to-end by minimizing the standard cross-entropy loss. To effectively decouple the complex task of graph structure discovery from spatio-temporal feature learning, a specialized two-stage training procedure is employed. This procedure is partitioned into a graph exploration phase and a subsequent model fine-tuning phase.

The initial phase, spanning the first *T*_*graph*_ epochs, is dedicated to graph exploration. During this stage, all network components, including the node embeddings *Z* within the RSGL and the STGCN encoder parameters, are optimized jointly. To identify a graph structure that promotes strong generalization, the model's performance is monitored on a validation set throughout this phase. The adjacency matrix A^*^ that corresponds to the lowest observed validation loss is preserved as the optimal graph.

In the second phase, the model transitions to fine-tuning. The optimal graph A^*^ is frozen, and the parameters of the RSGL are no longer updated. The network then continues training for the remaining epochs, with optimization focused exclusively on the STGCN and classifier parameters. This allows the feature extraction backbone to refine its capabilities on a stable, high-quality, and task-optimized graph structure.

This two-stage procedure provides a significant advantage by balancing the flexibility of adaptive graph learning with the stability required for robust feature extractor convergence. This methodology ultimately yields both superior classification performance and a more neuroscientifically interpretable final graph structure.

### 2.5 Computational complexity analysis

A computational analysis of the RS-STGCN framework is performed. This analysis evaluates the efficiency of the model. It considers both time and space complexity. The variables are the number of nodes *N* and the embedding dimension *D*. The final sparse graph has *E* edges. Other variables include batch size *B* and max sequence length *T*. The STGCN channels are *C*_*in*_ and *C*_*out*_. The temporal kernel is *K*_*t*_.

#### 2.5.1 Time complexity analysis

The overall time complexity is determined by two distinct phases. These are the graph learning phase and the spatio-temporal processing phase. The first stage is the RSGL graph learning. This stage executes once per epoch during the initial training phase. Its complexity is *O*(*N*^2^*D*) for the similarity matrix. The MST construction has a cost of *O*(*N*^2^). The TopK selection has a complexity of *O*(*N*^2^log*K*).

The second stage is the STGCN forward pass. This cost applies to the second training phase and inference. It operates on the sparse graph with *E* edges. The GCNConv operation has a complexity of *O*(*B*·*T*·*E*·*C*_*in*_·*C*_*out*_). The TCN operation has a complexity of *O*(*B*·*T*·*N*·*C*_*out*_·*K*_*t*_). The total per-batch time complexity *T* is:


$$\begin{array}{l} T\left(B,N,T\right)=O\left(B\cdot T\cdot \left(E\cdot C_{in}C_{out}+N\cdot C_{out}K_{t}\right)\right) \end{array}$$ (14)

#### 2.5.2 Space complexity analysis

The space complexity *S* is determined by the largest data structures. Model parameters are relatively small. The node embeddings require *O*(*ND*) storage. The graph learning phase stores a dense similarity matrix of *O*(*N*^2^). The STGCN forward pass stores intermediate activations. This cost is *O*(*B*·*C*_*out*_·*N*·*T*). The total space complexity is dominated by these two terms.


$$\begin{array}{l} S\left(B,N,T\right)=O\left(N^{2}+B\cdot C_{out}\cdot N\cdot T\right) \end{array}$$ (15)

The complexity analysis indicates a two-part cost. The RSGL module adds an *O*(*N*^2^) overhead. This cost applies only during the initial graph learning phase. The second training phase and all inference steps are highly efficient. Their time complexity scales linearly with the sparse edge count *E*. The cost does not scale with *N*^2^. This two-stage design makes the model practical for inference.

### 2.6 Experimental setup

#### 2.6.1 Datasets

The model's effectiveness was evaluated on two public EEG datasets. These were the SEED and SEED-IV datasets (Zheng and Lu, 2015; Zheng et al., 2019). The SEED dataset contains recordings from 15 subjects. It covers three emotion categories: positive, neutral, and negative. The SEED-IV dataset expands the task to four emotion categories. These categories are happy, sad, fear, and neutral. This expansion presents a more challenging classification benchmark. Both datasets utilize 62-channel EEG signals. These two datasets are widely recognized as the primary benchmarks for subject-independent emotion recognition. Their standard protocols facilitate rigorous comparison against state-of-the-art methods.

#### 2.6.2 Feature extraction and preprocessing

This study used the officially provided DE features. These features were extracted from five frequency bands. The bands included delta, theta, alpha, beta, and gamma. Each trial was structured as a tensor with dimensions for channels, time steps, and frequency bands. The time dimension was right-padded with zeros during batch processing. This ensured uniform length for model input. The labels for SEED and SEED-IV were mapped to integer values.

#### 2.6.3 Baseline models

The performance of the proposed model was benchmarked against a comprehensive set of competing methods. To ensure a fair and direct comparison, the results for these baseline models were drawn from prior studies. We only included results from publications that utilized the exact same subject-independent, Leave-One-Subject-Out (LOSO) evaluation protocol on the SEED and SEED-IV datasets. These baselines were categorized into three distinct groups. The first group comprised traditional machine learning algorithms such as STM (Chu et al., 2016), SVM (Suykens and Vandewalle, 1999), TCA (Pan et al., 2010), SA (Fernando et al., 2013), GFK (Gong et al., 2012), T-SVM (Collobert et al., 2006), KLIEP (Sugiyama et al., 2007), and ULSIF (Kanamori et al., 2009).

The second group included non-graph deep learning approaches. These ranged from DAN (Li H. et al., 2018) and DANN (Ganin et al., 2016) to advanced models like A-LSTM (Song et al., 2019), BiDANN-s (Li et al., 2018a), BiDANN (Li et al., 2018a), and BiHDM (Li et al., 2020). Finally, the third category featured state-of-the-art graph-based deep learning models. These were DGCNN (Song et al., 2018) and RGNN (Zhong et al., 2020). This extensive comparison provides a thorough evaluation against the existing literature.

#### 2.6.4 Model ablation design

The contribution of each key model component was evaluated through an ablation analysis. The analysis involved five ablated variants of the full model. The first variant excluded the temporal convolution module (“w/o TCN”). The second variant omitted the spatial graph convolution module (“w/o GCN”). To specifically assess the RSGL components, two further variants were designed. The “w/o Inter-TopK” variant removed all inter-regional connections, relying only on the intra-regional MSTs. The “w/o Intra-MST” variant removed the intra-regional backbones, relying only on the inter-regional TopK connections. The final variant excluded the Regional Spatio-Temporal Graph Learning module entirely (“w/o RSGL”). The performance of these variants was compared against the complete RS-STGCN model.

#### 2.6.5 Evaluation protocol

All experiments were performed under a subject-independent protocol. This is essential for evaluating the model's generalization capabilities across individuals. The primary evaluation metric was classification accuracy. It was assessed using LOSO cross-validation scheme. This necessary methodological constraint is essential to ensure a rigorous and direct comparison against the current state-of-the-art, as these are the standard benchmarks used by most SOTA models. In each LOSO fold, one subject was used for testing. The remaining subjects were used for training. The final accuracy was reported as the average across all folds. The number of active edges in the learned graph was also monitored. This provided insight into the model's structural learning process.

#### 2.6.6 Implementation details

The proposed model was implemented using the PyTorch framework. Graph convolutional operations were handled by PyTorch Geometric. The model was trained for 100 epochs using the Adam optimizer. The learning rate was set to 1.5e-3 with a batch size of 128. A dropout rate of 0.3 and a weight decay of 1e-4 were applied for regularization. The training process involved two stages. The graph structure was learned in the first 70 epochs. It was then frozen for fine-tuning in the final 30 epochs.

To ensure reproducibility, all experiments were conducted with a fixed random seed of 42, following standard practice. This parameter is defined at the beginning of the released code. The proposed RS-STGCN model was trained entirely from scratch without using any pre-trained weights. The model was implemented using PyTorch (version 2.1.0) and PyTorch Geometric (version 2.4.0). Key dependencies are detailed in the requirements.txt file in the code repository. All experiments were conducted on a workstation equipped with an AMD Ryzen 7 9800X3D 8-Core Processor CPU and an NVIDIA RTX 5080 GPU. The average training time for a single LOSO fold (100 epochs) was approximately 343.53 seconds.

## 3 Results

In this study, the performance of the proposed RS-STGCN model was evaluated using classification accuracy using a subject-independent LOSO approach. In each fold, one subject was randomly selected as the test set, and the remaining subjects were used as the training set.

### 3.1 Comparative performance on subject-independent classification

The performance of the proposed RS-STGCN model was evaluated against a comprehensive set of baseline methods. As detailed in Table 1, these baselines include traditional machine learning, deep learning, and state-of-the-art graph-based algorithms.

**Table 1:** Subject-independent classification accuracy (Mean/Std) on SEED and SEED-IV.

**Model**	**SEED**						**SEED-IV**
	**Delta band**	**Theta band**	**Alpha band**	**Beta band**	**Gamma band**	**All bands**	**All bands**
**Traditional machine learning models**							
STM	-	-	-	-	-	51.23/14.82	39.39/12.40
SVM	43.06/08.27	40.07/06.50	43.97/10.89	48.63/10.29	51.59/11.83	56.73/16.29	37.99/12.52
TCA	44.10/08.22	41.26/09.21	42.93/14.33	43.93/10.06	48.43/09.73	63.64/14.88	56.56/13.77
SA	53.23/07.47	50.60/08.31	55.06/10.60	56.72/10.78	64.47/14.96	69.00/10.89	64.44/09.46
GFK	-	-	-	-	-	71.31/11.49	64.38/11.41
T-SVM	-	-	-	-	-	72.53/14.00	-
KLIEP	-	-	-	-	-	45.71/17.76	31.46/09.20
ULSIF	-	-	-	-	-	51.18/13.57	32.99/11.05
**Non-graph deep learning models**							
DAN	-	-	-	-	-	83.81/08.56	58.87/08.13
DANN	-	-	-	-	-	75.08/11.18	47.59/10.01
BiDANN-S	63.01/07.49	63.22/07.52	63.50/09.50	73.59/09.12	73.72/08.67	84.14/06.87	65.59/10.39
BiDANN	-	-	-	-	-	83.28/09.60	65.59/10.39
BiHDM	-	-	-	-	-	85.40/07.53	69.03/08.66
A-LSTM	-	-	-	-	-	72.93/13.19	-
**Graph-based deep learning models**							
DGCNN	49.79/10.94	46.36/12.06	48.29/12.28	56.15/14.01	54.87/17.53	79.95/09.02	52.82/09.23
RGNN	64.88/06.87	60.69/05.79	60.84/07.57	74.96/08.94	77.50/08.10	85.30/06.72	73.84/08.02
**RS-STGCN (ours)**	**75.70/02.63**	**84.30/05.88**	**85.63/04.93**	**76.89/02.54**	**76.89/02.41**	**88.00/04.20**	**85.43/02.86**

As presented in the table, the RS-STGCN model demonstrated superior performance across both the SEED and SEED-IV datasets. Under the “all bands” condition, the model achieved a mean accuracy of 88.00% on SEED and 85.43% on SEED-IV. These results significantly surpass all other methods. Furthermore, the model exhibited exceptional stability. Its corresponding standard deviations of 4.20% and 2.86% were substantially lower than those of the next-best model, RGNN (6.72% and 8.02% respectively).

This performance advantage was also evident in the analysis of individual frequency bands on the SEED dataset. The model showed its most significant performance gains in the theta, alpha, and delta bands. For instance, its accuracy of 85.63% in the alpha band exceeded the top-performing baseline, RGNN (60.84%), by approximately 25%. Notably, the accuracy under the “all bands” condition surpassed the best performance achieved in any single frequency band.

### 3.2 Ablation study

An ablation study was conducted to evaluate the contribution of each core component of the RS-STGCN model. The performance of five model variants was compared against the complete model, with results presented in Table 2.

**Table 2:** Ablation study on core components of RS-STGCN.

**Variants**	**SEED**						**SEED-IV**
	**Delta band**	**Theta band**	**Alpha band**	**Beta band**	**Gamma band**	**All bands**	**All bands**
w/o TCN	66.96/01.11	66.67/00.00	65.78/03.33	63.56/08.02	64.74/07.61	68.00/03.12	66.22/06.03
w/o GCN	76.75/04.36	75.41/30.29	74.81/07.38	80.44/03.56	82.52/05.31	84.74/07.07	80.99/05.55
w/o Inter-TopK	76.00/03.16	75.85/2.42	74.96/03.39	83.26/05.79	82.52/06.07	86.52/05.23	79.51/02.98
w/o Intra-MST	75.41/02.87	75.41/04.17	74.52/02.42	80.59/03.29	82.81/06.17	84.00/06.65	77.53/04.96
w/o RSGL	76.15/02.63	75.70/02.63	74.96/03.93	79.56/03.16	82.37/04.69	83.70/07.73	78.77/04.96
**Complete model**	**76.89/02.54**	**76.89/02.41**	**75.70/02.63**	**84.30/05.88**	**85.63/04.93**	**88.00/04.20**	**85.43/02.86**

The removal of the temporal convolution network (“w/o TCN”) caused the most significant performance degradation. On the SEED dataset, its accuracy in the alpha band was 65.78%. On SEED-IV, its accuracy was 66.22%. The exclusion of the graph convolution network (“w/o GCN”) also reduced performance. The model's accuracy on SEED-IV dropped from 85.43% to 80.99%. Similarly, the model without the RSGL module (“w/o RSGL”) showed a notable decrease in accuracy, falling to 83.70% on SEED (all bands). The new variants, “w/o Inter-TopK” and “w/o Intra-MST,” achieved 86.52% and 84.00% respectively. These also underperformed the complete model. The complete model consistently achieved the highest accuracy across all conditions.

### 3.3 Sensitivity analysis of inter-regional budget

**Figure 2:**
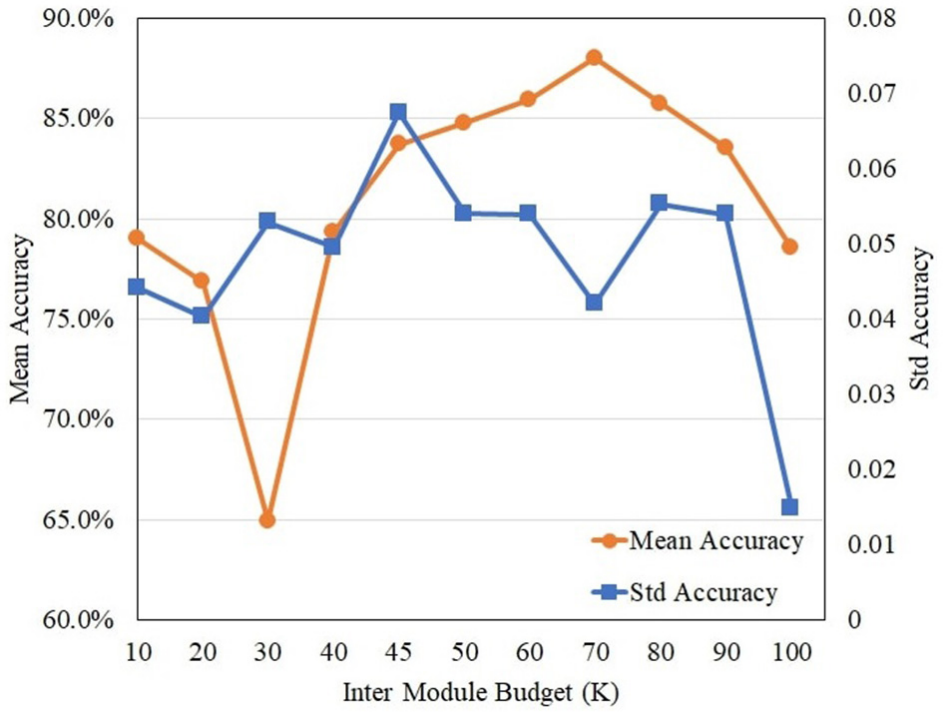
Sensitivity analysis of the inter-regional budget (*K*) on the SEED dataset.

An analysis was conducted to determine the optimal inter-regional connection budget (*K*). This hyperparameter controls the sparsity of the graph. The model's performance on the SEED dataset was evaluated across a range of K values. Figure 2 plots the resulting mean accuracy and standard deviation. The analysis identified *K* = 70 as the optimal value. This value achieved the peak mean accuracy (88.00%). This value was selected for all experiments in this study.

### 3.4 Visualization of the learned graph structure

**Figure 3:**
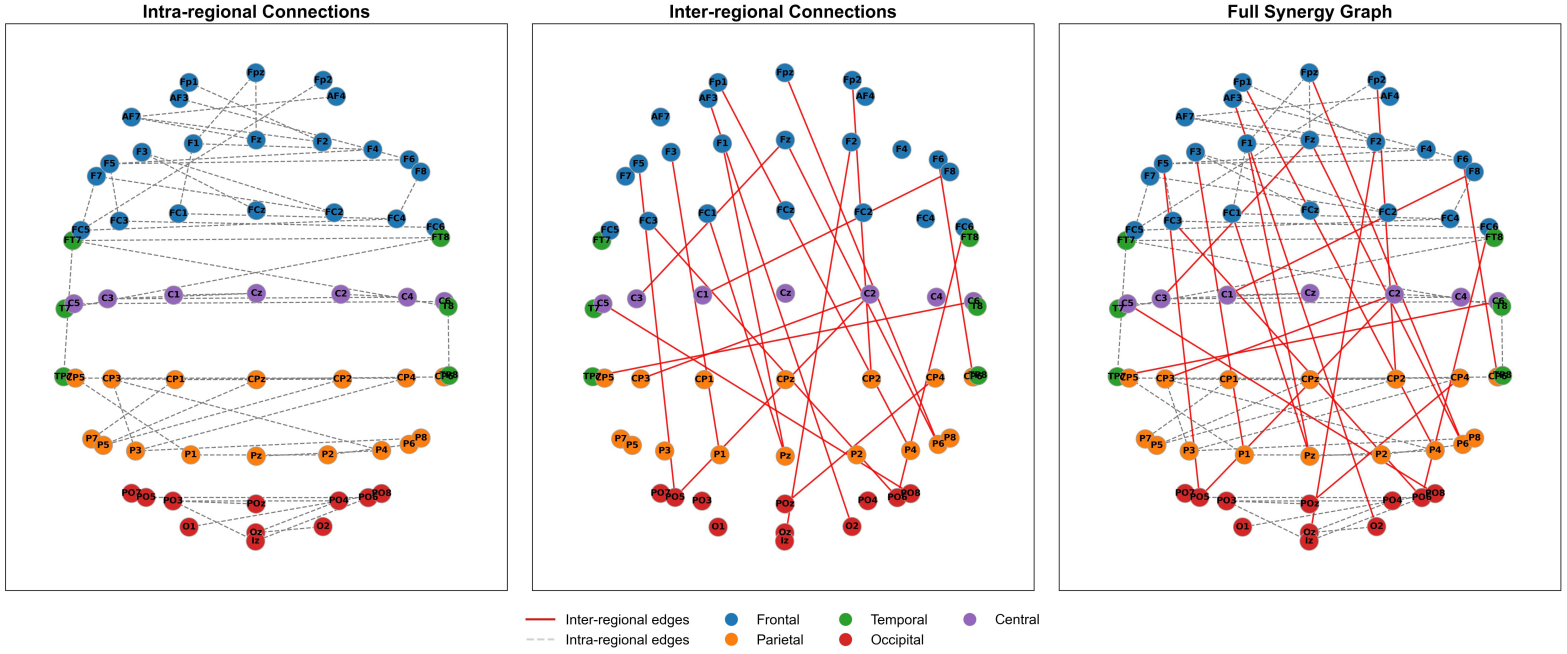
Decomposition of the regional synergy graph learned by RS-STGCN.

The functional connectivity patterns learned by the model were visualized. This provides neuroscientific interpretability for the RS-STGCN model. As presented in Figure 3, the nodes are arranged according to the 10–20 international system for EEG electrode placement. Different colors denote distinct brain regions. These are frontal (blue), temporal (green), central (purple), parietal (orange), and occipital (red).

The figure deconstructs the final graph into its constituent components. These components are the intra-regional connections, the inter-regional connections, and the full synergy graph. Intra-regional connections are depicted as dashed gray lines. They form a sparse backbone within each predefined brain region. Inter-regional connections are shown as solid red lines. They capture critical long-range dependencies identified by a Top-K mechanism. The integrated synergy graph thus balances biological plausibility with task-specific optimization.

## 4 Discussion

### 4.1 Analysis of comparative results

Classical machine learning models such as SVM and TCA are often limited by linear assumptions. They struggle to capture the complex, nonlinear dynamics inherent in EEG signals. Similarly, non-graph deep learning models like DAN and A-LSTM process EEG data as feature sequences. This approach disregards the brain's topological structure and known functional connectivity. While advanced models like BiDANN and BiHDM use attention to weigh key channels, they still treat electrodes as independent features. This overlooks the collaborative network nature of the brain, fundamentally constraining their performance.

The competitive performance of graph-based models like DGCNN and RGNN in Table 1 validates the importance of network-based analysis. However, these models are limited by their reliance on a static, predefined graph structure. This is where the advantages of RS-STGCN become evident, not only in mean accuracy but also in model stability.

A thorough analysis of Table 1 reveals the model's superior robustness. RS-STGCN achieves a significantly lower standard deviation on both SEED (4.20%) and SEED-IV (2.86%). This contrasts sharply with the high variance of top baselines like RGNN (8.02%) and BiHDM (8.66%) on SEED-IV. This low variance across subjects suggests that the RSGL's constrained, adaptive learning finds a more consistent and generalisable connectivity pattern. This pattern is less susceptible to the inter-subject variability that may affect other models.

Furthermore, the analysis of individual frequency bands provides deeper neuroscientific insights. The performance gains of RS-STGCN are most noticeably in the theta (84.30%) and alpha (85.63%) bands. This finding is crucial, as these bands are widely implicated in attentional control and emotional regulation. Static graphs may fail to capture the transient, task-specific functional connectivity occurring in these critical bands. The RSGL's task-driven optimisation successfully identifies and exploits these crucial connections, leading to a substantial performance increase.

Finally, the “all bands” condition (88.00%) surpasses the best single-band performance, which was 85.63% from the alpha band. This demonstrates that the model operates as an effective information integrator. RS-STGCN does not merely rely on one dominant frequency. Instead, it successfully fuses complementary information distributed across all five frequency bands. This confirms that the neural signature of emotion is a complex, multi-spectral pattern.

This robust, multi-spectral integration capability explains the model's superior generalization. This is particularly evident on the more challenging SEED-IV dataset. The model surpassed the top-performing baselines by substantial margins. It achieved an 11% improvement over RGNN and a 16% improvement over BiHDM. This large performance gap is not the only indicator of a robust improvement. The model's stability (2.86% std) is dramatically higher than that of both RGNN (8.02%) and BiHDM (8.66%). This combination of a large performance gap and significantly lower variance strongly implies the improvement is statistically significant and not a result of random chance. This margin and stability confirm the framework's ability to maintain high performance when faced with more complex, multi-class emotion tasks.

### 4.2 Impact of key model components

The ablation study results confirm that each component of the RS-STGCN framework provides a distinct and crucial contribution. The significant performance drop in the “w/o TCN” variant underscores the importance of temporal convolutions. These are essential for capturing the dynamic dependencies inherent in multi-channel EEG signals over time.

Furthermore, the reduced accuracy of the “w/o GCN” variant validates the necessity of modeling spatial dependencies. Graph convolutions effectively capture cross-channel relationships, which are overlooked by non-graph methods. The performance decrease in the “w/o RSGL” variant confirms the overall benefit of adaptive graph learning. The component-level ablations provide deeper insights. Both the “w/o Inter-TopK” and “w/o Intra-MST” variants underperformed the complete model. This result demonstrates that neither component is sufficient alone.

Notably, on the more complex SEED-IV dataset, the “w/o Intra-MST” variant (77.53%) performed worse than the “w/o RSGL” baseline (78.77%). This suggests that relying solely on sparse inter-regional connections without the stable intra-regional backbones can be detrimental to generalization. The model's success is not from simply adding these components, but from their synergistic interaction.

Beyond mean accuracy, the standard deviation across subjects offers deeper insights into model robustness. The “w/o GCN” variant revealed a critical instability. Its standard deviation in the theta band surged to 30.29%. This suggests that without graph convolutions, the model fails to find consistent emotional patterns in the theta band across different subjects. The result implies that emotion-related information in the theta band is heavily encoded in the inter-channel spatial relationships. GCN provides an essential mechanism to robustly extract this relational information, thereby stabilizing performance. This finding reinforces the critical role of spatial modeling for reliable EEG-based emotion recognition.

The superior performance and stability of the complete model demonstrate that these components work synergistically to capture complex spatio-temporal brain dynamics.

From an empirical perspective, the proposed RS-STGCN exhibits the smallest standard deviation of classification accuracy among all compared models, demonstrating its stability and reproducibility across LOSO folds. This consistent performance supports that the two-stage training and the deterministic MST-based intra-regional graph jointly contribute to a robust and generalisable learning process.

### 4.3 Analysis of inter-regional sparsity

The sensitivity analysis in Figure 2 provides key insights. It confirms the importance of the inter-regional connection budget (*K*). The model's performance is highly sensitive to this parameter. When *K* is too low (e.g., 30), the mean accuracy drops significantly to 64.94%. This suggests the model fails to capture necessary long-range dependencies for emotional integration. Conversely, when K is too high (e.g., 100), accuracy also degrades to 78.52%. This indicates that too many connections may introduce spurious edges. These spurious edges act as noise and harm the model's generalization. The analysis revealed an optimal trade-off at *K* = 70. This value is sparse enough to prevent noise. It is also dense enough to capture critical long-range functional connections.

### 4.4 Neuroscientific interpretation of learned connectivity

The learned graph provides a neuroscientifically interpretable map of functional brain connectivity during emotion recognition. Notably, the most prominent inter-regional links emerge between the frontal and occipital lobes, and between the frontal and parietal lobes. The strong frontal–occipital synchronization may reflect enhanced phase coherence between these regions, a phenomenon observed in recent EEG studies (Chen et al., 2022). Concurrently, the robust frontal-parietal network mirrors top-down attentional control mechanisms. This involves executive regions modulating sensory areas to facilitate emotion recognition (Veniero et al., 2021; Wu et al., 2022).

The structure of the synergy graph reveals a key insight into modeling complex systems like the brain. The model does not learn a uniform graph. Instead, it discovers a synergistic interplay between two types of spatio-temporal relationships. The sparse, intra-regional connections represent stable, physiologically plausible local information processing. In contrast, the targeted, inter-regional connections represent flexible, task-driven global information integration. This balance is a significant finding. It demonstrates that the model adaptively identifies a connectivity pattern optimized for the specific cognitive task, a feat unattainable by methods relying on predefined static graphs.

Recent EEG-based graph learning studies have adopted similar interpretative strategies to connect learned graph structures with established neuroscientific evidence. For instance, EEG-GNN (Demir et al., 2021), EEG-GAT (Demir et al., 2022), and EEG-GCNN (Wagh and Varatharajah, 2020) analyse the spatial organization of learned connectivity patterns to highlight functional relationships among brain regions. Following these studies, the present work interprets the learned inter- and intra-regional connections by comparing them with well-documented neural pathways such as the frontal–parietal and frontal–occipital networks associated with attentional and emotional regulation. This alignment with prior neuroscientific findings supports the biological plausibility and interpretability of the learned graph.

These data-driven findings suggest a potential neural strategy underlying emotion recognition. The strategy may involve an initial top-down modulation of visual features via frontal-occipital pathways. This is followed by attentional integration through frontal-parietal pathways, culminating in emotional judgment. This interpretation aligns with established frameworks of attentional control and emotion-cognition interaction. It demonstrates that RS-STGCN effectively learns functionally meaningful and interpretable brain network representations, offering a powerful tool for analyzing spatio-temporal dynamics in other complex cognitive systems.

In summary, this study proposed the RS-STGCN, a novel framework for EEG-based emotion recognition. We demonstrated that the RSGL module, by modeling brain connectivity at two synergistic levels, resolves a key conflict between static priors and unconstrained graph learning. The framework achieved state-of-the-art, generalisable performance on the SEED and SEED-IV datasets. It also produced neuroscientifically interpretable connectivity maps, identifying key pathways consistent with established cognitive networks.

Despite these contributions, limitations exist. The RSGL currently relies on predefined brain regions based on electrode locations. Future investigations might explore methods to learn these functional parcels directly from the data, rather than relying on a fixed anatomical prior. Additionally, the framework was validated on emotion recognition in healthy subjects. Applying the RS-STGCN framework to identify biomarkers for clinical affective disorders, such as depression or anxiety, presents a valuable and impactful direction for future research.

## Data Availability

Publicly available datasets were analyzed in this study. This data can be found here: the public datasets analyzed for this study can be found at the following locations. The SEED dataset is available at: https://bcmi.sjtu.edu.cn/home/seed/seed.html. The SEED-IV dataset is available at: https://bcmi.sjtu.edu.cn/home/seed/seed-iv.html.
